# Ultrafast Isolation of Synaptic Terminals From Rat Brain for Cryo-Electron Tomography Analysis

**DOI:** 10.21769/BioProtoc.5429

**Published:** 2025-09-05

**Authors:** Rong Sun, Qiangjun Zhou

**Affiliations:** Department of Cell and Developmental Biology, Vanderbilt Brain Institute, Center for Structural Biology, Vanderbilt Kennedy Center, Vanderbilt University, Nashville, TN, USA

**Keywords:** Synaptosome, Synaptoneurosome, Cryo-electron tomography, Synaptic terminal isolation, Hippocampus, Rat brain

## Abstract

Understanding the nanoscale organization and molecular rearrangement of synaptic components is critical for elucidating the mechanisms of synaptic transmission and plasticity. Traditional synaptosome isolation protocols involve multiple centrifugation and resuspension steps, which may cause structural damage or alter the synaptosomal fraction, compromising their suitability for cryo-electron tomography (cryo-ET). Here, we present an ultrafast isolation method optimized for cryo-ET that yields two types of synaptosomal fractions: synaptosomes and synaptoneurosomes. This streamlined protocol preserves intact postsynaptic membranes apposed to presynaptic active zones and produces thin, high-quality samples suitable for in situ structural studies. The entire procedure, from tissue homogenization to vitrification, takes less than 15 min, offering a significant advantage for high-resolution cryo-ET analysis of synaptic architecture.

Key features

• Ultrafast synaptic terminal isolation from tissue homogenization to vitrification completed within 15 min.

• Retention of postsynaptic membranes with synaptic receptors and postsynaptic density (PSD) proteins.

• The thickness of the samples is suitable for in situ cryo-ET analysis.

• Enables cryo-ET studies of synaptic structures and postsynaptic membrane proteins such as AMPA receptors.

## Graphical overview


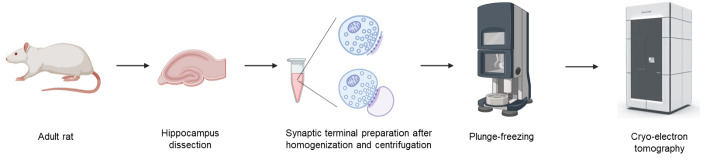


## Background

Cryo-electron tomography (cryo-ET) allows visualization of 3D cellular structures in their native states at nanometer resolution [1]. Synaptic elements, which are critical for neurotransmission, are particularly challenging to image with cryo-ET due to their sample thickness and molecular complexity. Isolated synaptic terminals, including synaptosomes and synaptoneurosomes, are used as a model to study synaptic structure and physiology [2,3]. Traditional methods for synaptosome or synaptoneurosome preparation often involve multiple centrifugation and resuspension steps, which might damage delicate structures and increase preparation time [4]. By streamlining the process to dissection, homogenization, and a single-step centrifugation, this protocol significantly reduces sample processing time and preserves ultrastructure, enabling direct cryo-ET studies of synaptic organelles and proteins, including synaptic vesicles, adhesion molecules, postsynaptic receptors, and synaptic nanoblocks in the postsynaptic density (PSD). Specifically, we quantitatively analyzed the distribution of tethered, docked, and partially fused synaptic vesicles, which indicates the presynaptic potential release sites, as well as the distribution of postsynaptic receptors and synaptic nanoblocks [5]. These results demonstrate that synaptic terminals isolated using our protocol are suitable for cryo-ET and subsequent quantitative analyses of synaptic components.

## Materials and reagents

**Biological materials**

1. 10-week-old Sprague-Dawley rats of either sex (Charles River Laboratories)

**Reagents**

1. Isoflurane (Piramal, catalog number: NDC 66794-017-25)

2. Sucrose (Sigma-Aldrich, catalog number: S9378)

3. HEPES (Fisher BioReagent, catalog number: BP310-1)

4. EDTA (Millipore, catalog number: 324503)

5. 10-nm BSA-gold beads (Aurion, catalog number: 25486)

6. NaOH (Fisher, catalog number: AC259860010)

7. Ethane gas (A-L Compressed Gases, catalog number: AGETHANECP-7)

8. Liquid nitrogen (A-L Compressed Gases, catalog number: GSINL240)

**Solutions**

1. 0.5 M EDTA stock solution (see Recipes)

2. 10-nm gold beads solution (see Recipes)

3. Iso-osmotic homogenization buffer (see Recipes)

**Recipes**

**1. 0.5 M EDTA stock solution**

To prepare 0.5 M EDTA (pH 8.0), dissolve 93 g of EDTA disodium salt in ~400 mL of deionized water in a 500 mL autoclavable screw-cap bottle with a magnetic stir bar. The EDTA will not dissolve until the pH is adjusted. Slowly add ~10 g of NaOH pellets while stirring until the pH reaches 8.0. Once fully dissolved, transfer the solution to a 500 mL graduated cylinder, adjust to the final volume with deionized water, remove the stir bar, and return the solution to the bottle. Autoclave and store at room temperature.

**2. 10-nm gold beads solution**

To prepare a 10 nm BSA-gold solution, centrifuge 300 μL of BSA-conjugated gold nanoparticles at 16,100× *g* for 30 min at 4 °C. Carefully discard the supernatant without disturbing the pellet. Resuspend the pellet gently in 150 μL of iso-osmotic homogenization buffer (Recipe 3) by pipetting or brief vortexing. Store the solution at 4 °C and use within a week. 10-nm gold beads are added to the sample when plunge freezing. These gold beads will be used as fiducial markers to align the tilt series during reconstruction.

**3. Iso-osmotic homogenization buffer**

Prepare 500 mL of homogenization buffer as a 0.32 M sucrose solution by adding 54.77 g of sucrose in ~400 mL of deionized water and stirring in a 500 mL autoclavable screw-cap bottle with a magnetic stir bar. This is then supplemented with 0.477 g of HEPES and 1 mL of 0.5 M EDTA stock (Recipe 1) while stirring until dissolved, and finally adjusted to pH 7.4.

**Laboratory supplies**

1. Quantifoil R2/2 Cu 200 mesh EM grids (Quantifoil)

2. Cryo grid box (SubAngstrom, catalog number: SBV01)

## Equipment

1. Small animal decapitator (Fisher Scientific, catalog number: 10-000-124)

2. Overhead stirrer (Electron Microscopy Sciences, catalog number: 6480610)

3. 10-mL Teflon head tissue grinder (Electron Microscopy Sciences, catalog number: 6479310)

4. Centrifuge (Eppendorf, model: 5415D)

5. FEI Vitrobot Mark III (FEI, now Thermo Fisher Scientific)

6. Titan Krios G4 Cryo-EM (Thermo Fisher Scientific)

7. K3 camera (Gatan)

## Software and datasets

1. Thermo Fisher Tomography 5 https://www.thermofisher.com/us/en/home/electron-microscopy/products/software-em-3d-vis/tomography-software.html

2. IMOD https://bio3d.colorado.edu/imod/ [6]

3. MotionCorr2 https://emcore.ucsf.edu/ucsf-software [7]

4. Gctf https://github.com/JackZhang-Lab/GCTF [8]

## Procedure

**A. Synaptic terminal isolation**

1. Deeply anesthetize 10-week-old rats with isoflurane.

2. Dissect hippocampal tissues in ice-cold homogenization buffer. Our dissection procedure was modified from a previously described method [9], as follows: Following anesthesia, the rat was euthanized using a small animal decapitator. The fur and skull were quickly removed to expose the brain, which was then immediately extracted and transferred to a Petri dish containing ice-cold homogenization buffer, which must be kept on ice throughout the procedure. The hippocampi were then carefully isolated from both hemispheres.

3. Weigh the hippocampal pair and put the tissue into a 10-mL Teflon head tissue grinder. Add 10 mL/g of ice-cold homogenization buffer.

4. Homogenize tissues using an overhead stirrer at ~500 rpm for four strokes. Each stroke is about 1–2 s.

5. Centrifuge the homogenate at 800× *g* for 10 min at 4 °C.

6. Collect the supernatant immediately for cryo-EM grid preparation. Carefully pipette the supernatant without disturbing the pellet at the bottom. Keep the supernatant on ice until use.

**B. Isolated synaptic terminal vitrification**

1. Set Vitrobot Mark III parameters as follows: 100% humidity, 4 °C, blot time 3.5 s, blot total 1, blot offset -1.5, wait time 6 s.

2. Mix the supernatant with 10-nm gold beads solution at a 1:1 ratio.

3. Apply 4 μL of mixture onto Quantifoil R2/2 Cu 200 mesh EM grids.

4. Blot and plunge grids into liquid ethane cooled by liquid nitrogen.

5. Store vitrified grids in liquid nitrogen until use.

**C. Cryo-ET data collection**

**Figure 1.**
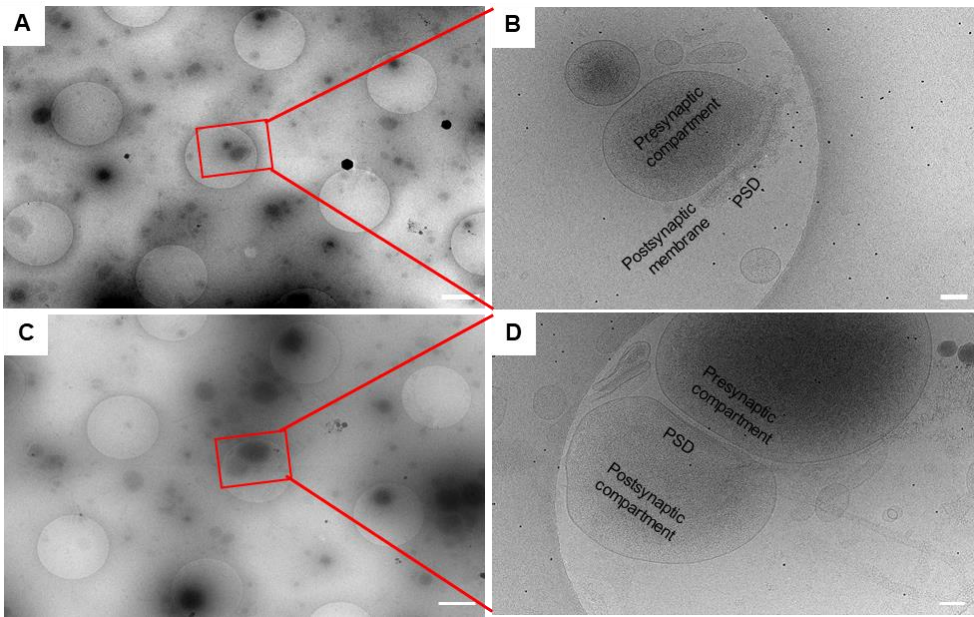
Isolated synaptic terminals imaged at different magnifications. (A, C) Low-magnification (4,800×) search images acquired at a pixel size of 3.69 nm. Scale bars, 1 μm. (B, D) High-magnification (26,000×) exposure images acquired at a pixel size of 3.3 Å. Scale bars, 100 nm. In the synaptosome (B), the enclosed presynaptic compartment, postsynaptic membrane, and postsynaptic density (PSD) are visible. In the synaptoneurosome (D), the enclosed presynaptic compartment and postsynaptic compartment, as well as the PSD, are clearly identifiable.

1. Load vitrified grids into Titan Krios G4 equipped with a Gatan K3 camera.

2. Use batch tomography acquisition in Tomography 5 software.

3. Search for synaptosome or synaptoneurosome structures at search magnification 4,800× with the pixel size of 3.69 nm. The features are recognizable at this magnification. Search for enclosed presynaptic compartments with synaptic vesicles and postsynaptic membranes with PSD for potential synaptosomes (Figure 1A). Search for enclosed presynaptic compartments with synaptic vesicles and enclosed postsynaptic compartments with PSD for potential synaptoneurosomes (Figure 1C).

4. Set up batch tomography positions where there are potential synaptosomes or synaptoneurosomes. Refine all positions before collection. The refinement process will take low-dose exposure images of all positions. Double-check the high magnification exposure images to make sure the picked positions are synaptosomes (Figure 1B) or synaptoneurosomes (Figure 1D) with certain features as described in step C3.

5. Collect tilt series using voltage phase plate: magnification 26,000×, pixel size 3.3 Å, defocus -1 to -2 μm, dose-symmetric scheme from 0° to ±60° at 2° intervals.

## Data analysis

**Figure 2.**
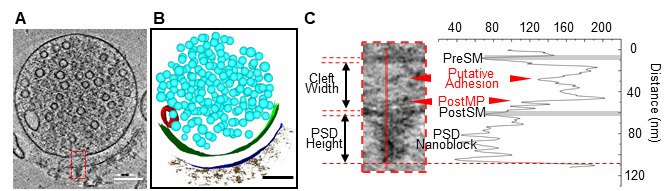
Ultrastructural analysis of an excitatory synaptosome. (A) Tomographic slice showing an excitatory synaptosome. (B) 3D segmentation of the synaptosome shown in panel A. (C) Quantification of the synaptic cleft and the postsynaptic density (PSD) shown in panel A. The zoomed-in tomographic slice showing the excitatory synaptosome from panel A is on the left, and its density plot showing gray values along the red solid line is on the right. Scale bars (A, B), 100 nm. PreSM, presynaptic membrane. PostMP, postsynaptic membrane protein. PostSM, postsynaptic membrane.

1. Perform motion correction (MotionCorr2) and contrast transfer function (CTF) estimation (Gctf).

2. Align tilt series and reconstruct tomograms using IMOD.

3. Segment synaptosomes using IMOD and identify synaptic structural features (Figure 2).

## Validation of protocol

This protocol was validated by structural studies of synaptic terminals, including identification of potential presynaptic release sites, postsynaptic nanoblocks, and receptor-like particles, as described in Sun et al. [5] "The postsynaptic density in excitatory synapses is composed of clustered, heterogeneous nanoblocks," Journal of Cell Biology, 2025 (Figures 1–3, 5, Supplemental Figures 1 and 3, and Video 2).

## General notes and troubleshooting

This protocol employs a single-step centrifugation following homogenization to remove large debris. As a result, the frozen sample contains not only synaptosomes and synaptoneurosomes but also various smaller cellular fragments. The primary challenge lies in identifying synaptosomes or synaptoneurosomes among these structures within the low-magnification search maps covering the EM grid squares. We typically found 1–3 synaptosomes and synaptoneurosomes per grid square. Careful screening of each grid square is essential. With patience and persistence, synaptic terminals can be reliably located.
